# A new animal model of placebo analgesia: involvement of the dopaminergic system in reward learning

**DOI:** 10.1038/srep17140

**Published:** 2015-11-25

**Authors:** In-Seon Lee, Bombi Lee, Hi-Joon Park, Håkan Olausson, Paul Enck, Younbyoung Chae

**Affiliations:** 1 Acupuncture and Meridian Science Research Center, College of Korean Medicine, Kyung Hee University, Seoul, Korea; 2 Department of Internal Medicine: Psychosomatic Medicine and Psychotherapy, University of Tübingen, Tübingen, Germany; 3 Institute for Medical Psychology, fMEG Center, University of Tübingen, Tübingen, Germany; 4 IMPRS for Cognitive and Systems Neuroscience, University of Tübingen, Tübingen, Germany; 5 Center for Social and Affective Neuroscience, Linköping University

## Abstract

We suggest a new placebo analgesia animal model and investigated the role of the dopamine and opioid systems in placebo analgesia. Before and after the conditioning, we conducted a conditioned place preference (CPP) test to measure preferences for the cues (Rooms 1 and 2), and a hot plate test (HPT) to measure the pain responses to high level-pain after the cues. In addition, we quantified the expression of tyrosine hydroxylase (TH) in the ventral tegmental area (VTA) and c-Fos in the anterior cingulate cortex (ACC) as a response to reward learning and pain response. We found an enhanced preference for the low level-pain paired cue and enhanced TH expression in the VTA of the Placebo and Placebo + Naloxone groups. Haloperidol, a dopamine antagonist, blocked these effects in the Placebo + Haloperidol group. An increased pain threshold to high-heat pain and reduced c-Fos expression in the ACC were observed in the Placebo group only. Haloperidol blocked the place preference effect, and naloxone and haloperidol blocked the placebo analgesia. Cue preference is mediated by reward learning via the dopamine system, whereas the expression of placebo analgesia is mediated by the dopamine and opioid systems.

Recent research on the mechanism of placebo analgesia has examined the brain’s response to painful stimulation modulated by expectation or Pavlovian conditioning. Early studies in humans demonstrated that administration of a neurotransmitter antagonist (naloxone) reversed the placebo-induced analgesic effect^1^,^2^. Subsequent studies have investigated differential or overlapping mechanisms between conditioning and the expectation-induced placebo effect, including the endogenous opioid, cannabinoid, and dopamine systems^3^,^4^,^5^. Furthermore, the development of neuroimaging techniques has helped researchers to determine the brain mechanism of placebo analgesia in humans. Several studies have shown that activity is altered due to expectation-6 or conditioning-induced placebo analgesia^7^ via a μ-8 or non-opioid^9^ mechanism in brain regions associated with pain processing and pain modulation (e.g., the frontal cortex, cingulate cortex, insula).

Placebo analgesia was first investigated in humans^10^. In human studies, verbal instructions and subjective ratings are important in inducing and measuring the phenomenon, and the mechanisms of placebo analgesia may differ due to disease, physical and emotional conditions, therapeutic interventions, the medical environment, and the manner in which the placebo is induced^11^. A desire to maximize placebo responses in clinical practice^12^ and interest in drug–placebo interactions^13^ have been discussed in recent studies showing the important role of cognitive factors on the placebo effect in experimental and clinical settings in human. Although human studies also successfully contributed to reveal the mechanism of placebo effect, there is still a strong need for an animal model of placebo analgesia^14^,^15^,^16^,^17^. First, it is generally hard to control for these cognitive factors in human studies because they include a sheer magnitude of elements such as context, beliefs, culture, previous experiences, prejudices, expectations, etc^18^. The animal model has advantages over human studies in terms of controlling previous experience and environmental factors, drug administration, brain lesion or genetic modification, and measurement of pain-related neurochemicals in the brain directly^19^. Existing animal models employ Pavlovian conditioning paradigms (e.g., injection of an analgesic drug as a conditioning cue) and measure behavioral pain responses. However, the injection procedure is painful, which could lead to stress responses that cause emotional and physical changes in the animal^20^,^21^, and the active drug injection could also interact with placebo-specific changes in the animals. The placebo analgesia studies in animals could be improved by examining the placebo analgesia related neurotransmitter activities in the brain.

To overcome the limitations of previous studies and investigate the specific mechanism underlying placebo analgesia, we used healthy rats and a conditioning paradigm in which a neutral cue was conditioned with different pain intensities. We also introduced a differentiation between the learning phase (learning of low-pain-paired (LPP) and high-pain-paired (HPP) cues are important phases that precede placebo analgesia in rodents^4^,^22^,^23^,^24^) from the placebo analgesia response (expression) phase. The major benefit of our experimental paradigm is that the placebo analgesia related brain activities in the learning (acquisition) and the placebo analgesia response (expression) phase could be measured separately. Dopamine plays an important role in Pavlovian conditioning^25^, and reward learning^26^; and haloperidol (dopamine antagonist) blocks the place preference induced by conditioning with various drugs (methamphetamine, cocaine, amphetamine, morphine, etc.). Moreover, as opioids are involved in placebo analgesia, naloxone (opioid antagonist) blocks the opioid conditioning-induced or expectation-induced placebo analgesia in both experimental and clinical pain models^1^,^2^,^3^,^8^,^14^,^27^. Here we used Pavlovian conditioning of neutral cues with heat pain stimulation, and hypothesized that the dopamine system was involved in the cue-learning (acquisition) phase and that the endogenous opioid system was involved in the analgesia response (expression) phase of placebo analgesia.

To examine the role of dopamine and opioid systems in two phases of placebo analgesia, rats were divided into four groups: Control, Plaecbo, Placebo + Naloxone, and Placebo + Haloperidol groups. Immunoreactivity was analyzed in dopamine or opioid related brain areas, ventral tegmental area (VTA) and anterior cingulate cortex (ACC), and behavioral tests were conducted to assess the degree of cue learning and the placebo analgesia.

## Methods

### Animals

Adult male Sprague-Dawley rats (n = 82) weighing 220–230 g (7 weeks old) were obtained from Samtako Animal Co. (Seoul, Korea). The animals were housed in a limited-access rodent facility with up to five rats per polycarbonate cage and maintained on a 12:12 h day-night cycle (lights on from 8.00 a.m. to 8.00 p.m.) at room temperature (23 ± 0.5 °C). Sterilized drinking water and a standard chow diet were supplied ad libitum to each cage, and the animals’ weights were measured to monitor their physical condition. All experiments began at least 5 days after the animals arrived. The animal experiments were conducted in accordance with the National Institutes of Health (NIH) Guide for the Care and Use of Laboratory Animals (NIH Publications no. 80–23, revised in 1996), and were approved by the Kyung Hee University Institutional Animal Care and Use Committee.

### Experimental design

Rats were excluded if they: 1) were hypersensitive (a hind paw withdrawal latency [HPWL] of less than 50 s to 45 °C) or hyposensitive (an HPWL of more than 20 s to 50 °C) to thermal pain in the heat pain sensitivity test (n = 8); 2) spent more than 300 s at the Center (33.3% of the total time) during conditioned place preference (CPP) test 1 (n = 18); 3) did not prefer either of the two cues in CPP test 1 (unbiased rats, n = 13); or 4) had malformed feet (n = 1). After exclusion, rats were divided into four groups: Control (n = 10), Placebo (n = 16), Placebo + Naloxone (P + N; n = 8), and Placebo + Haloperidol (P + H; n = 8).

The groups, drug administration, and conditioning cues and pain stimuli are described in Table 1. Placebo analgesia was induced in the three placebo groups (Placebo, P + N, and P + H) by placebo conditioning (experiencing low level-pain after Room 1 and high level-pain after Room 2), whereas rats in the Control group experienced an identical number of cues and heat pain in the absence of conditioning (both low- and high-heat pain after both cues). To specify the underlying mechanism in the acquisition and expression of placebo analgesia, naloxone or haloperidol (Sigma-Aldrich Chemical Co., St Louis, MO, USA) was administered via intraperitoneal injection (i.p.; P + N or P + H group, respectively) before the CPP and hot plate tests (HPTs). Antagonists (naloxone and haloperidol) were dissolved in saline and injected at a concentration of 5 and 0.02 mg/kg, respectively. The concentrations were determined based on previous rodent studies that showed significant blockage of placebo analgesia or CPP without adverse events^14^,^17^. Animals in the Placebo and Control groups received an i.p. saline injection instead of an antagonist injection. Considering the stability of learning processes and pharmacokinetic phases, the injection time was set to 15 min before CPP test 2 and 30 min before HPT 2.

Tyrosine hydroxylase (TH) is an enzyme that catalyzes the amino acid L-tyrosine to L-3,4-dihydroxyphenylalanine (L-DOPA), a dopamine precursor. c-Fos is used as an indicator of the neuronal response to pain^28^. We examined the expression of TH in the ventral tegmental area (VTA) and c-Fos in the anterior cingulate cortex (ACC) using immunohistochemistry.

### Apparatus

#### CPP apparatus

**Figure 1:**
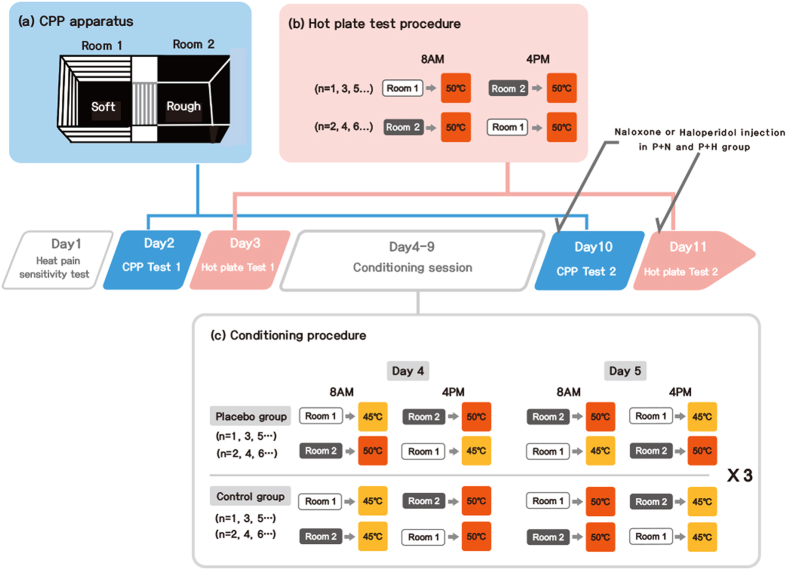
Experimental procedure. Heat pain sensitivity test (Day 1), conditioned place preference (CPP) test 1 (Day 2), hot plate test (HPT) 1 (Day 3), conditioning sessions (Days 4–9), CPP test 2 (Day 10), HPT 2 (Day 11). (**a**) The CPP tests were conducted in a CPP apparatus including Room 1 (black and white striped wall with soft floor) and Room 2 (black wall with rough floor). During the CPP test, the doors were raised to 10 cm above the floor, and the rats explored the CPP apparatus for 15 min. This test measured the rats’ preferences for Rooms 1 and 2. (**b**) The HPTs were conducted to measure the placebo analgesic effect; rats were exposed to a 50 °C stimulus for 1 min after being exposed to Rooms 1 and 2 for 15 min. The procedure was identical for each of the four groups, and the hind paw withdrawal latencies (HPWLs) were measured. (**c**) The guillotine doors were closed during the conditioning sessions. Rats were located in Room 1 or Room 2 for 15 min, and were exposed to 45 °C or 50 °C for 1 min (2 sessions per day for 6 days). As the results of CPP test 1 showed that the rats preferred Room 2 to Room 1, Room 1 was chosen as the non-preferred room and paired with low level-pain and Room 2 was paired with strong pain in the Placebo groups (Placebo, Placebo + Naloxone [P + N], Placebo + Haloperidol [P + H]). There was no fixed pairing between the cue and pain intensity in the Control group. The order was counterbalanced throughout the experiment. CPP: conditioned place preference, n: number, P + H: Placebo+Haloperidol, P + N: Placebo + Naloxone.

A CPP apparatus (San Diego Instruments, San Diego, CA) was used during CPP tests and the conditioning sessions. The apparatus had three compartments (left and right ends of Rooms 1 and 2, 26.37 × 20.65 × 33.35 cm; Center, 15.88 × 20.65 × 33.35 cm); photo beams detected the amount of time that the rats spent in each compartment (Fig. 1). The three compartments were divided with manually operated guillotine doors. Rooms 1 and 2 had walls and floors of different shapes (visual aspect of the cue) and textures (tactile aspect of the cue). Room 1 had horizontal black and white striped walls and a soft black floor; Room 2 had black walls with a rough black floor^29^. The Center had white walls with a bar-shaped wire floor. The brightness of each room was adjusted by ceiling LED lights set to 7 lux for Rooms 1 and 2 and 30 lux for the Center^30^. The whole apparatus was covered by a black curtain and maintained at room temperature (25 °C) during the experiments.

#### HPT apparatus

Heat pain was delivered with an HPT apparatus (Bioseb, Chaville, France), which had a 16.5 × 16.5 cm metal plate that could reach 65 ± 0.5 °C. It had transparent acrylic walls that were 45.5 cm in height. The HPT was located close to the CPP apparatus (25 °C room temperature).

### Procedures

#### Heat pain sensitivity test

Before the conditioning session, we conducted three baseline tests: heat pain sensitivity test, CPP test 1, and HPT 1. After 4 days of adaptation, rats were allowed to explore the CPP and HPT apparatus freely (hot plate set at 25 °C) for 15 min to habituate. The following day (Day 1), the heat pain sensitivity test was administered using 45 °C and 50 °C stimuli. The temperature was chosen based on pilot experiments, and a 1 min duration was chosen to prevent tissue damage. There were 8 h between the two tests (one started at 8.00 a.m. and other at 4.00 p.m.), and the order was counterbalanced. All rats were placed in their home cage immediately after testing. Experiments were recorded and the HPWL was measured.

#### Measurement of cue preference changes with the CPP test

Two CPP tests (CPP test 1 and CPP test 2, before and after the conditioning session) were conducted and the results were compared to investigate changes in cue preference on Days 2 and 10 (Fig. 1). On Day 2, CPP test 1 was performed at 8.00 a.m. to measure the baseline preferences for Rooms 1 and 2. Rats were positioned on the starting point (Center) and freely passed into Room 1 or 2 for 15 min (both guillotine doors opened). On Day 10, 15 min after the drug (or saline) injection, CPP test 2 was performed in a manner similar to CPP test 1.

#### Measurement of placebo analgesia with the HPT

Two HPTs (HPT 1 and HPT 2, before and after the conditioning session, respectively) were conducted to compare the HPWL response to high-heat pain after the cues (Fig. 1). Rats were placed in Room 1 for 15 min, and immediately thereafter experienced high level-pain for 1 min; then the procedure was repeated in Room 2. One session started at 8.00 a.m. and the other at 4.00 p.m., and the order was counterbalanced. Naloxone or haloperidol was administered to the P + N or P + H group via injection 30 min before HPT 2 on Day 11 (post-conditioning test).

#### Conditioning sessions

Conditioning was accomplished over 2 sessions per day for 6 days (Days 4–9, 12 sessions in total; Fig. 1). In one session, all rats in the Placebo groups were placed in Room 1 (LPP) for 15 min (guillotine doors closed), and then immediately placed at 45 °C (low level pain) for 1 min. In the other session, rats were placed in Room 2 (HPP) for 15 min, and then placed at 50 °C (high level-pain) for 1 min (placebo conditioning paradigm). Rats in the Control group experienced four different pairs of cues and pain intensity (45 °C after Room 1, 50 °C after Room 1, 45 °C after Room 2, 50 °C after Room 2, three times, respectively). One session started at 8.00 a.m. and other at 4.00 p.m., and the order was counterbalanced.

#### TH and c-Fos immunohistochemistry

Rats were deeply anesthetized using sodium pentobarbital (80 mg/kg, i.p.), and then perfused through the ascending aorta with saline (0.9%), followed by 300 mL 4% paraformaldehyde in 0.1 M phosphate-buffered saline (PBS). The brains were removed, post-fixed overnight, and cryoprotected with a 20% sucrose solution in 0.1 M PBS at 4 °C. Coronal sections (20 μm thick) were cut through the VTA and ACC using a cryostat (Leica CM1850; Leica Microsystems Ltd., Nussloch, Germany). The sections were collected from the respective brain areas as determined according to the Paxinos and Watson rat brain atlas^31^. Briefly, the sections were rinsed three times for 5 min each in PBS, and then incubated with a rabbit anti-c-Fos (1:2,000 dilution; Chemicon International Inc., Temecular, CA, USA) or sheep anti-TH antibody (1:2,000 dilution; Chemicon International Inc.) in PBS containing 0.3% Triton X-100 (PBST) for 72 h at 4 °C. The sections were washed for 5 min in PBS and then incubated for 2 h at room temperature with a biotinylated goat anti-rabbit immunoglobulin (Ig)G secondary antibody (for the anti-c-Fos antibody) or a biotinylated goat anti-sheep IgG secondary antibody (for the anti-TH antibody). The sections were incubated for 120 min at room temperature with the secondary antibodies. Both secondary antibodies were obtained from Vector Laboratories Co. (Burlingame, CA, USA) and diluted 1:200 in PBST containing 2% normal goat serum. To visualize the immunoreactivity, the sections were incubated for 90 min in the avidin-biotin complex (ABC) reagent (Vectastain Elite ABC kit; Vector Labs CO., Burlingame, CA, USA), washed three times for 5 min each in PBS, and incubated in a solution containing 3,3′-diaminobenzidine (DAB; Sigma-Aldrich Chemical Co.,) and 0.01% H_2_O_2_ for 1 min. Finally, the tissues were washed in PBS, followed by a brief rinse in distilled water, and individually mounted onto slides. Slides were allowed to air dry, and then were cover-slipped. Images were captured using an Olympus BX53 digital microscope and cellSens digital imaging software (Olympus American, Inc., Center Valley, PA, USA). The sections were viewed at 100× magnification, and the number of cells within 450 × 450 μm^2^ grids was counted. The cells within the tissues were counted in at least four different sections in four rats in each group.

### Statistical analysis

The results are expressed as the mean ± standard error (SE). Coefficient values were calculated to measure the changes in CPP and HPWL between the pre- and post-conditioning tests^32^. This minimizes the individual differences by excluding the time spent in the Center room in CPP test and calculate the relative preference or pain response before and after the conditioning as follows: CPP coefficient = (Time spent in Room 1 – Time spent in Room 2)/(Time spent in Room 1 + Time spent in Room 2) ×100. The HPT coefficient was calculated using the HPWL to high level-pain after Room 1 or Room 2 as follows: HPT coefficient = (HPWL to 50 °C after Room 1 – HPWL to 50 °C after Room 2)/(HPWL to 50 °C after Room 1 + HPWL to 50 °C after Room 2) × 100. Placebo analgesia was defined as an increase in the HPT coefficient after conditioning. CPP and HPT coefficients were analyzed with paired *t*-tests between the pre- and the post-conditioning tests in each group using SPSS (version 13.0; SPSS, Inc., Chicago, IL, USA). Spearman correlation analyses were used to determine the changes in CPP and HPT coefficients in the Placebo group.

Immunohistochemical data were analyzed using a one-way analysis of variance (ANOVA) followed by Tukey’s post-hoc tests in SPSS. A significance level of P < 0.05 was used for all analyses.

## Results

### Heat pain sensitivity test

The HPWL to the 50 °C stimulus was 11.9 ± 0.7 s. No rats withdrew their hind paws to the 45 °C stimulus.

### Cue preference changes in the CPP test

**Figure 2:**
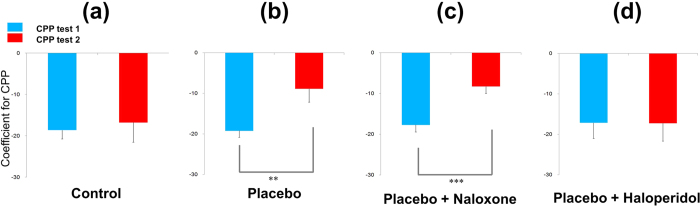
The CPP coefficient value indicated the relative preference between Rooms 1 and 2 (low-pain-paired [LPP] vs. high-pain-paired [HPP] cues in the Placebo groups, non-paired cues in the Control group). The CPP coefficient = (Time spent in Room 1–Time spent in Room 2)/(Time spent in Room 1 + Time spent in Room 2) × 100. The preference for cues between the pre- and post-conditioning tests did not significantly differ in the Control group. In the Placebo group, the non-preferred cue (Room 1) from the pre-conditioning test was paired with low level-pain in the conditioning session; the rats’ preference for the LPP cue significantly increased after conditioning (*p* < 0.01). Increased preference to the LPP cue was also significant in the P + N group (*p* < 0.001), but not in the P + H group. Values are presented as means ± standard errors (SEs). CPP: conditioned place preference, P + H: Placebo+Haloperidol, P + N: Placebo + Naloxone.

Relative cue preferences (coefficient of CPP test 1) did not significantly differ among the four groups before the conditioning (blue bar in Fig. 2). The CPP test 1 coefficient value was −18.6 ± 2.2 for the Control group and −19.2 ± 1.6 for the Placebo group. For CPP test 2 (after conditioning), the coefficient value was −16.8 ± 4.7 for the Control group and −8.9 ± 3.3 for the Placebo group (Fig. 2a,b). The CPP test 1 and 2 coefficient values did not significantly differ in the Control group (Fig. 2a), whereas the CPP coefficient value significantly increased in the Placebo group after placebo conditioning (−19.2 ± 1.6 vs. −8.9 ± 3.3, *t* = −3.04, *p* < 0.01). These findings indicate an increase in the preference for Room 1 (LPP cue) compared to Room 2 (HPP cue) (Fig. 2b).

The coefficient value of CPP test 1 was −17.7 ± 1.7 for the P + N group and −17.1 ± 3.9 for the P + H group. The coefficient value of CPP test 2 was −8.3 ± 1.7 for the P + N group and −17.2 ± 4.4 for the P + H group (Fig. 2c,d). The CPP coefficient significantly increased in the P + N group (−17.7 ± 1.7 vs. −8.3 ± 1.7, *t* = −6.01, *p* < 0.001; Fig. 2c); however, there were no significant changes in the P + H group. This suggests that the increase in the preference to Room 1 (LPP cue) after placebo conditioning was blocked after haloperidol administration (Fig. 2d).

### Placebo analgesia with the HPT

**Figure 3:**
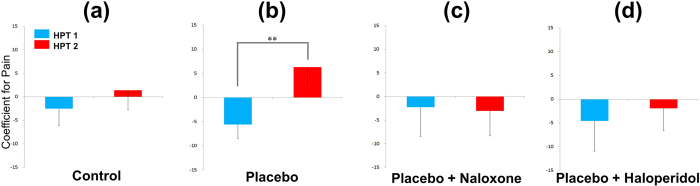
The HPWL coefficient value revealed the relative pain response to strong pain after Rooms 1 and 2. HPT coefficient = (HPWL to 50 °C after Room 1–HPWL to 50 °C after Room 2)/(HPWL to 50 °C after Room 1 + HPWL to 50 °C after Room 2) × 100. In the Control group, there were no significant differences in the HPWL coefficient value for the 50 °C stimulus after the cues between the pre- and post-conditioning tests. In the Placebo group, the coefficient value significantly increased in the post-conditioning test compared to the pre-conditioning test (placebo analgesia, *p* < 0.01). Placebo analgesia (increased HPWL coefficient compared to the pre-conditioning test) was not observed in the P + N or P + H groups. Values are presented as means ± SEs. HPT: hot plate test, HPWL: hind paw withdrawal latency, P + H: Placebo + Haloperidol, P + N: Placebo+Naloxone.

The pain response after the cues (coefficient of HPT 1) did not significantly differ among the four groups before conditioning (blue bar in Fig. 3). The coefficient value of HPT 1 was −2.5 ± 3.6 for the Control group and −5.6 ± 6.3 for the Placebo group; the coefficient value of HPT 2 was 1.4 ± 4.1 for the Control group and 6.3 ± 3.9 for the Placebo group (Fig. 3a,b). There was a significant increase in the HPT coefficient in the Placebo group (−5.6 ± 6.3 vs. 6.3 ± 3.9, *t* = −3.106, *p* < 0.01; Fig. 3b), but not in the Control group. This indicates that the HPWL to high level-pain after Room 1 compared to the HPWL to high level-pain after Room 2 increased when Room 1 was paired with low level-pain (placebo analgesic effect).

The coefficient value of HPT 1 was −2.3 ± 6.2 in the P + N group and −4.5 ± 6.4 in the P + H group. The coefficient value of HPT 2 was −3.1 ± 5.3 in the P + N group and −1.9 ± 4.7 in the P + H group (Fig. 3c,d). The coefficients for HPT 1 and HPT 2 did not significantly differ in the P + N and P + H groups, suggesting a lack of placebo analgesia after naloxone or haloperidol injection.

### Correlation analysis between cue preference and placebo analgesia

There was a significant correlation (*r* = 0.521, *p* < 0.05) between the changes in coefficient values for CPP and HPT (before and after the conditioning), which demonstrated a correlation between the cue preference and placebo analgesia.

### TH-like immunoreactivity in the VTA

**Figure 4:**
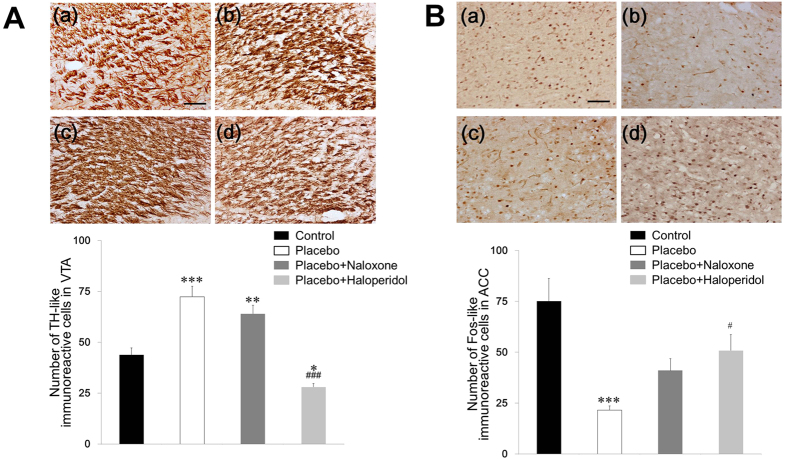
Effect of placebo conditioning and antagonist administration on tyrosine hydroxylase (TH) in the ventral tegmental area (VTA) and c-Fos expression in the anterior cingulate cortex (ACC). (**A**) TH expression in the VTA of the Control (**a**), Placebo (**b**), P + N (**c**), and P + H groups (**d**). (**B**) c-Fos expression in the ACC of the Control (**a**), Placebo (**b**), P + N (**c**), P + H groups (**d**). Values are presented as the mean ± SE of the total number of TH or Fos-like immunoreactive neurons within a 450 × 450 μm^2^ grid over the areas at 100× magnification. *p < 0.05, **p < 0.01, ***p < 0.001 versus the Control group and ^#^*p* < 0.05, ^###^*p* < 0.001 versus the Placebo group. The scale bar represents 100 μm. P + H: Placebo+Haloperidol, P + N: Placebo + Naloxone.

The number of TH-like immunoreactive cells in the VTA was 43.8 ± 3.4 in the Control group, 72.4 ± 5.1 in the Placebo group, 64.0 ± 4.2 in the P + N group, and 28.0 ± 1.7 in the P + H group. The number of TH-like immunoreactive cells significantly differed among the groups (ANOVA followed by Tukey’s post-hoc tests, F_(3, 12)_ = 36.152, *p* < 0.001). We observed significantly more TH-like immunoreactive cells in the VTA region of the Placebo (*p* < 0.001) and P + N groups (*p* < 0.01) than in the Control group. There were significantly fewer TH-like immunoreactive cells in the P + H group than in the Control (*p* < 0.05) and other Placebo groups (*p* < 0.001), which demonstrated the successful blockage of TH expression in the VTA region (Fig. 4A).

### Fos-like immunoreactivity in the ACC

In the ACC, the number of Fos-like immunoreactive cells was 75.0 ± 11.2 in the Control group, 21.5 ± 2.0 in the Placebo group, 41.0 ± 5.8 in the P + N group, and 50.8 ± 7.8 in the P + H group. The number of Fos-like immunoreactive cells significantly differed among the groups (ANOVA followed by Tukey’s post-hoc tests, F_(3, 12)_ = 12,070, *p* < 0.001). There were significantly fewer Fos-like immunoreactive cells in the ACC region of the Placebo group than in the Control group (*p* < 0.001). There were significantly more Fos-like immunoreactive cells in the ACC in the P + H and P + N groups than in the Placebo group (Fig. 4B).

## Discussion

Rats displayed a significantly increased pain threshold when the pain was delivered after the LPP cue, indicating placebo analgesia. The enhanced preference for the LPP cue and the enhanced expression of TH in the VTA of placebo-conditioned rats were blocked by a dopamine antagonist but not by an opioid antagonist. The reduced pain response and c-Fos expression in the ACC were blocked by both dopamine and opioid antagonists. These results indicate that the dopamine system is involved in both cue learning and placebo analgesia, whereas the endogenous opioid system is involved in the analgesic response phase but not in the cue learning phase.

Learning and previous experiences play important roles in human placebo response^11^,^33^. In this study, we separately analyzed the cue learning and placebo analgesia expression phases. Haloperidol, a D2, D3, and D4 dopamine receptor antagonist, prevented TH activities in the VTA, preferences for the LPP cue, and placebo analgesia. This may indicate that impaired cue learning due to reduced dopamine activity disrupted the placebo analgesia. However, both reward learning and aversive learning (preference for the LPP cue and avoidance of the HPP cue) are associated with the cue learning phases. Expectation of low level-pain produces placebo analgesia, whereas expectation of high level-pain produces a *nocebo* effect^34^. Further research is necessary to distinguish the role of dopamine and brain activity in reward and avoidance learning^35^ and the placebo and nocebo effects in placebo analgesia^36^. Recently Wrobel *et al.* carried out a neuroimaging study and reported that the haloperidol administered in healthy human participants had no significant effect on placebo analgesia^37^. The discrepant results might have been found due to more complex learning and pain processing networks in the human brain compared to rodents such as the involvement of higher association cortex. In the study of Wrobel *et al.*, the prefrontal cortex and secondary somatosensory cortex were reported as the main brain areas associated in the placebo analgesia. Moreover, as Wrobel *et al.* have pointed out already, the dose of haloperidol as well as the detailed differences in the methods of the conditioning paradigm (temperature of stimuli, number of sessions, days, etc.) might have been critical for the different findings.

On the other hand, naloxone, a μ-opioid receptor antagonist, prevented the placebo analgesia effect but did not affect the preference for the LPP cue. Thus, for placebo analgesia, opioids might affect pain processing per se rather than interrupting the learning process. These results also demonstrated the involvement of opioids in neutral cue conditioning, in addition to their previously known role in expectation or opioid conditioning^3^,^14^,^38^. However, it is difficult to discriminate expectation- or conditioning-induced placebo analgesia, as opioids are also related with the reward^39^,^40^. Thus, further study of the role of opioids in neutral cue conditioning-induced placebo analgesia is required.

Traditionally, a neutral cue (conditioned stimulus, CS) can produce a conditioned response (CR) after conditioning with an unconditioned stimulus (US). In previous studies, a repeated US (drug application) paired with a contextual cue (CS, injection, or other cues: olfactory, visual, tactile, etc.) was used to establish a placebo analgesia animal model. These models were used to investigate whether the CS could relieve the pain (placebo analgesia, CR) in the absence of an US. Bryant *et al.*^41^ examined the analgesic effect after conditioning with an opioid receptor agonist. Other studies^14^,^15^,^16^ focused on placebo analgesia via vehicle injection after conditioning of active drugs in healthy rodents or in neuropathic pain models. Naloxone was the only antagonist used in previous studies^14^,^15^. One of the most important advantages of studies using naïve animals is the ability of the experimenter to control the variables, including cognitive, emotional, environmental, and other placebo triggers. However, experimental environments contain unintended contextual cues, such as the experimenter, handling, the environment, temporal arrangement, and injection during drug delivery^42^. In addition, saline injection, which was used in previous placebo studies, produces stress-related behavioral and physiological changes in rats^20^,^21^ that could affect the learning process and place preference^43^,^44^,^45^,^46^. Analgesic drugs could also interact with the neuronal/biochemical pathways of the placebo analgesia. Therefore, we used neutral cues with a manipulated pain level rather than injection cues and active drugs. Similar experimental designs (i.e., placebo cream or visual cue paired with low-heat pain) were used in human studies^6^,^37^. To the best of our knowledge, this is the first placebo analgesic experiment to employ neutral cue conditioning with manipulated pain intensity in animals. Furthermore, we used a combination of cues (visual and tactile) to make the cues more distinguishable, and considered the pain intensity, temporal contiguity, frequency, duration, and number of sessions for successful conditioning^47^,^48^.

The advantages of our model include: 1) being safer and less stressful compared to injection; 2) using a pain intensity that can be regulated depending on the subjects; 3) having two phases of placebo analgesia (acquisition and expression); and 4) being simple, which makes it possible to change or add details for further study. However, the efficacy, reliability, and long-term sustainability must be determined.

There were a number of limitations in this study. First, we did not include a natural history group, such as a no conditioning group. However, the control group in this study was exposed to an identical number of cues and similar heat pain to test changes in cue preference and pain response. Secondly, since we did not include a group that received haloperidol after the CPP phase, prior to the HPT, we cannot fully determine whether the abolished placebo effect in the haloperidol group was derived from the lack of learning or from the inhibition of dopamine release during the placebo test. In order to fully dissociate the mechanisms between dopamine and opioids during placebo analgesia, it is necessary to investigate the role of dopamine between the acquisition phase and the expression phase in placebo analgesia, which we plan to do in our future study. Furthermore, this study lacked subjective ratings, which are known to be more sensitive to the placebo effect than objectively measured outcomes^49^. Alternatively, in addition to behavioral data, we analyzed immunoreactivity in the brain, which could be a neuronal biomarker of placebo analgesia. The procedure in this study was simple and sufficient to produce placebo analgesia; however, if placebo analgesia can be produced in less than 12 sessions, the whole procedure could be shortened. Our animal model could be a useful experimental paradigm for further investigating the underlying neural mechanisms of placebo analgesia, including the role of conditioning cues (e.g., visual, olfactory, light, sound, tactile), environment and context, treatment ritual, and interventions (drug or non-pharmacological). Last but not least, haloperidol, a non-selective dopamine receptor antagonist, is known to act on various subtypes of dopaminergic receptors. In order to further explore the role of specific dopamine receptors, it is necessary to investigate the mechanisms of placebo analgesia also using other receptor-specific antagonists.

In summary, we demonstrated that conditioning a neutral cue with low or high level-pain resulted in a significant cue preference to the LPP cue and placebo analgesia in animals. A dopamine antagonist blocked the acquisition of cue preference by learning; both dopamine and opioid antagonists blocked the expression of placebo analgesia. The increased preference to the LPP cue was significantly correlated with placebo analgesia. As the placebo animal model in this study used a neutral cue-conditioning paradigm with manipulated pain, this study could stimulate further cue-, context-, and intervention-specific studies of placebo analgesia.

## Additional Information

**How to cite this article**: Lee, I.-S. *et al.* A new animal model of placebo analgesia: involvement of the dopaminergic system in reward learning. *Sci. Rep.* **5**, 17140; doi: 10.1038/srep17140 (2015).

## Figures and Tables

**Table 1:**
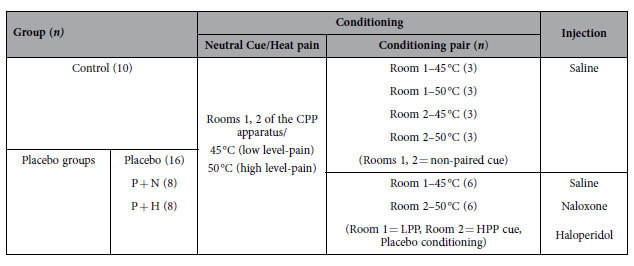
Conditioning paradigm in the Control and Placebo groups.

CPP: conditioned place preference, HPP: high-pain-paired, LPP: low-pain-paired, *n*: number, P + H: Placebo + Haloperidol, P + N: Placebo + Naloxone.
